# Genetic Parameter Estimation and Genome-Wide Association Study-Based Loci Identification of Milk-Related Traits in Chinese Holstein

**DOI:** 10.3389/fgene.2021.799664

**Published:** 2022-01-28

**Authors:** Xubin Lu, Abdelaziz Adam Idriss Arbab, Ismail Mohamed Abdalla, Dingding Liu, Zhipeng Zhang, Tianle Xu, Guosheng Su, Zhangping Yang

**Affiliations:** ^1^ College of Animal Science and Technology, Yangzhou University, Yangzhou, China; ^2^ Center for Quantitative Genetics and Genomics, Aarhus University, Tjele, Denmark; ^3^ Joint International Research Laboratory of Agriculture and Agri-Product Safety, Yangzhou University, Yangzhou, China

**Keywords:** Chinese holstein, milk-related traits, test-day model, genetic parameters, genome-wide association study (GWAS)

## Abstract

Accurately estimating the genetic parameters and revealing more genetic variants underlying milk production and quality are conducive to the genetic improvement of dairy cows. In this study, we estimate the genetic parameters of five milk-related traits of cows—namely, milk yield (MY), milk fat percentage (MFP), milk fat yield (MFY), milk protein percentage (MPP), and milk protein yield (MPY)—based on a random regression test-day model. A total of 95,375 test-day records of 9,834 cows in the lower reaches of the Yangtze River were used for the estimation. In addition, genome-wide association studies (GWASs) for these traits were conducted, based on adjusted phenotypes. The heritability, as well as the standard errors, of MY, MFP, MFY, MPP, and MPY during lactation ranged from 0.22 ± 0.02 to 0.31 ± 0.04, 0.06 ± 0.02 to 0.15 ± 0.03, 0.09 ± 0.02 to 0.28 ± 0.04, 0.07 ± 0.01 to 0.16 ± 0.03, and 0.14 ± 0.02 to 0.27 ± 0.03, respectively, and the genetic correlations between different days in milk (DIM) within lactations decreased as the time interval increased. Two, six, four, six, and three single nucleotide polymorphisms (SNPs) were detected, which explained 5.44, 12.39, 8.89, 10.65, and 7.09% of the phenotypic variation in MY, MFP, MFY, MPP, and MPY, respectively. Ten Kyoto Encyclopedia of Genes and Genomes pathways and 25 Gene Ontology terms were enriched by analyzing the nearest genes and genes within 200 kb of the detected SNPs. Moreover, 17 genes in the enrichment results that may play roles in milk production and quality were selected as candidates, including *CAMK2G*, *WNT3A*, *WNT9A*, *PLCB4*, *SMAD9*, *PLA2G4A*, *ARF1*, *OPLAH*, *M*GST1, *CLIP1*, *DGAT1*, *PRMT6*, *VPS28*, *HSF1*, *MAF1*, *TMEM98*, and *F7*. We hope that this study will provide useful information for in-depth understanding of the genetic architecture of milk production and quality traits, as well as contribute to the genomic selection work of dairy cows in the lower reaches of the Yangtze River.

## Introduction

The production and quality of milk are key factors that influence the profitability of dairy enterprises. Most milk-related traits, including the milk yield (MY), milk fat percentage (MFP), milk fat yield (MFY), milk protein percentage (MPP), and milk protein yield (MPY), are quantitative traits, which are controlled by multiple genes and are sensitive to environmental influences (Wang et al., 2019a). Understanding the genetic architecture, estimating the genetic parameters, and revealing more quantitative trait loci (QTL) regions underlying these milk-related traits are beneficial to the genetic improvement of dairy cows, as the genetic variation information could be utilized more rationally and effectively.

The reliability of genetic parameter estimation is one of the important factors affecting population genetic improvement. The random regression test-day model has been widely applied to the genetic evaluation of traits that are measured repeatedly at different time points, as it simulates the environmental and genetic effects along the lactation trajectory, and provides higher accuracy (with a 6%–8% increase) than genetic evaluation based on the lactation model (using full or extended 305-day lactation records) in cows (Li et al., 2020). In addition, it is convenient to infer the total variance components of performance during the entire lactation period based on the variance components estimated from test-day records, without requiring individuals to have a record every day of the lactation period (Wahinya et al., 2020). Due to the goodness-of-fit and the low correlation among parameters, Legendre polynomials (LP)—especially the high-order polynomials—have generally been used to model the lactation curve of cows (Silvestre et al., 2006; Bignardi et al., 2009a). However, when estimating variance components (genetic parameters) using LP of the order higher than three, the further improvement becomes small, and the model generally has difficulty in converging (Li et al., 2020). Therefore, the appropriate selection of the LP order can improve the calculation efficiency and the goodness-of-fit, thus, resulting in a high accuracy of the genetic parameter evaluation in the test-day model (Pereira et al., 2013; Li et al., 2020).

A genome-wide association study (GWAS) can effectively identify the potential genetic variants that are associated with quantitative traits, and can also facilitate molecular breeding of animals (Hayes and Goddard, 2010). Previous studies have reported that the QTLs detected by GWASs are helpful in improving the accuracy of whole-genome prediction (Zhang et al., 2014), and constructing the genomic kinship matrix by all the causative quantitative trait nucleotides (QTNs) could maximize the genomic prediction accuracy (Fragomeni et al., 2017). Therefore, the discovery of more QTLs that affect milk-related traits could provide support for the genetic selection progress of dairy cows.

Chinese Holsteins are the crossbred progeny of the imported Holstein bulls from Europe and North America and the native yellow cattle breeds, thus, having complicated genetic background (Li et al., 2017). It has been reported that approximately 13% of Chinese Holstein blood comes from local breeds, and a considerable number of Chinese Holsteins are descendants of native Chinese cattle breeds (Liu et al., 2014; Liu et al., 2020). A study has revealed that the Chinese Holstein cattle population from Beijing showed a higher level of haplotype diversity than those in other regions, and part of Holstein bulls in Qinghai Province were crossed with the local yellow cattle and yak to improve milk production of the local breeds (Ferreri et al., 2011). Another study has also shown that the Chinese Holstein cows from southern China are more adapted to the hot and humid climate than the cows from northern China (Liu et al., 2014). Therefore, the proper genetic evaluation of dairy cows in different regions is necessary.

The lower reaches of the Yangtze River experience a temperate climate, with warm springs, hot and rainy summers, cool autumns, and relatively cold winters for the latitude (Ding et al., 2017). The cows in this region are susceptible to mastitis and heat stress in summer, as well as cold stress in winter, resulting in performance differences of milk-related traits, compared with farms in other areas (Yang et al., 2019). Among previous GWASs on the milk-related traits of the Chinese Holstein cows, most of the research populations were in the west or north of China (Jiang et al., 2010; Liu et al., 2020), and there have been very few association studies or estimation of genetic parameters on the milk-related traits based on the random regression test-day model focused on Chinese Holstein cows in the lower reaches of the Yangtze River. In this study, we estimated the genetic parameters of MY, MFP, MFY, MPP, and MPY of Chinese Holstein cows in the lower reaches of the Yangtze River, and conducted a GWAS on these traits. We expect that the estimates of genetic variance, as well as the newly identified genetic variants, may contribute to the genomic selection and genetic improvement of dairy cows in the lower reaches of the Yangtze River.

## Materials and methods

### Ethical Statement

This research was carried out in strict compliance with the Institutional Animal Care and Use Committee of the School of the Yangzhou University Animal Experiments Ethics Committee [Permit Number: SYXK(Su)IACUC 2012–0029], in the process of sample collection and data collection, and no animals felt uncomfortable or experienced malnutrition during this research.

### Research Population and Phenotype Collection

The research population in this study was 15,216 Holstein cows from four farms in Jiangsu Province, China, and 149,065 test-day records of these cows over 9 years (2010–2019) were collected. All of the records were measured by the Nanjing Dairy Cattle Performance Measurement Center (Nanjing, China). Five traits evaluating the milk performance of dairy cows were selected for genetic parameter evaluation and association analyses; namely, MY, MFP, MFY, MPP, and MPY. The phenotypic quality control of each cow was carried out following the criteria that the test-day records were from parity 1 to parity 3, the first calving age was between 22 and 36 months, the DIM was between 5 and 305, the number of records in a parity was greater than 6, and the milk production ranged from 5 to 80 kg in each record. After quality control, 9,834 Holstein cows and 95,375 test-day records from them were retained for the subsequent analysis. These cows were the offspring of 599 bulls. The pedigrees of these cows were traced to at least three generations, and were used to estimate genetic parameters of the traits with a random regression test-day model. The summary statistics of the phenotypes are presented in Table 1, and the distribution and correlation of the phenotypes are shown in Supplementary Figure S2.

**TABLE 1:** Descriptive statistics of test-day records and estimated heritability of 305-day performance of the milk-related traits in the study population.

Traits	N	Mean	SD	Median	Min	Max	Skew	Kurtosis	h^2^ (SE)
MY (kg)	95,375	33.33	6.23	33.00	5.00	80.00	0.31	0.43	0.34 (0.04)
MFP (%)	92,763	3.88	1.00	3.82	0.30	9.99	0.52	1.46	0.29 (0.05)
MFY (kg)	92,763	1.29	0.50	1.23	0.60	5.74	0.93	2.00	0.27 (0.02)
MPP (%)	92,763	3.27	0.37	3.25	0.30	9.46	0.75	4.95	0.32 (0.07)
MPY (kg)	92,763	1.09	0.32	1.07	0.10	4.58	0.36	1.02	0.28 (0.09)

Note. N, number of records; SD, standard deviation; Min, minimum; Max, maximum; h^2^, the total heritability of 305-day performance; SE, standard error.

### Genotypic Data

Among the 9,834 cows that passed the phenotypic quality control, hair samples from the tails of 999 cows were collected for DNA extraction and genotyping. The genotyping work was conducted at Neogen Biotechnology Co., Ltd. (MI, United States) using GGPBovine 100K SNP Chips, and the ARS-UCD 1.2 assembly of the *Bos taurus* genome was used as the reference genome. In total, 95,256 variants were derived from the 30 chromosomes of each cow. All of the variants were filtered, according to the following quality control standards: Call rate of variant >90%, minor allele frequency (MAF) >0.95, and Hardy–Weinberg Equilibrium (HWE) >1.0 × 10^−6^. In addition, it required that the call rate of individual genotypes be >0.95 (Marees et al., 2018). Finally, 984 cows and 84,407 variants passed the quality control, and were used in the association analysis.

### Estimated Genetic Parameters

A random regression test-day model was used for estimation of variance components of the milk-related traits using the DMU software (Madsen et al., 2006). In this model, we considered the herd-test days, parties, and first calving ages as fixed effects, the functions of DIM as fixed regression effects, and the individual additive genetic effects and individual permanent environmental effects as the random regression effects (Liu et al., 2020). The first calving ages of dairy cows were divided into four levels (age ≤23 months; 24 ≤ age ≤ 27; 28 ≤ age ≤ 31; and age ≥ 32). The DIM effect was fitted by a sixth-order Legendre polynomial, and the individual additive genetic effects and individual permanent environmental effects were fitted by a third-order Legendre polynomial (Li et al., 2020). The random regression test-day model was as follows (Li et al., 2020):

$$\begin{array}{c} y_{ijklmn}=HTD_{i}+PA_{j}+FCA_{k}+\sum_{n=0}^{6}b_{ln}L_{n}\left(\omega_{t}\right)+\sum_{n=0}^{3}a_{mn}L_{n}\left(\omega_{t}\right)+\sum_{n=0}^{3}p_{mn}L_{n}\left(\omega_{t}\right)+e_{ijklmn} \end{array},$$ (1)

where the $y_{ijklmn}$ is the phenotypic observation of the test-day record, $HTD_{i}$ is the fixed effect of the i th herd-test day, $PA_{j}$ is the fixed effect of the j th party, $FCA_{k}$ is the fixed effect of the k th level of first calving age (k = 1, 2, 3, and 4), $b_{ln}$ is the *n*th fixed regression coefficient on the *n*th Legendre polynomial, $a_{mn}$ is the n th random regression coefficient of the additive genetic effect of the m th cow, $p_{mn}$ is the *n*th random regression coefficient of the permanent environmental effect of the m th cow, $L_{n}\left(\omega_{t}\right)$ is the n th covariate of Legendre polynomial at day t in milk (DIM_t_), $\omega_{t}$ is the normalized time value at DIM_t_ (DIM = 5, 6, … , 305), and $e_{ijklmn}$ is the random residual. The residuals were assumed to be homogeneous throughout the whole lactation (Li et al., 2020). The model was required for convergence at the criterion that the norm of the update vector was less than 1.0 × 10^−7^ or the norm of the gradient vector (AI) was less than 1.0 × 10^−6^ (Madsen et al., 2006). The kinship matrix of animals was constructed considering the pedigree.

After removing the fixed effects, all traits were calibrated to the 305-day performance and were then used as the adjusted phenotypes for the subsequent association analysis. The adjustment process of each trait was as follows:

$$\begin{array}{c} y_{adj}=\sum_{t=5}^{305}\sum_{n=0}^{3}a_{mn}L_{n}\left(\omega_{t}\right)+\sum_{t=5}^{305}\sum_{n=0}^{3}p_{mn}L_{n}\left(\omega_{t}\right)+\sum_{t=5}^{305}e_{t} \end{array},$$ (2)

where the $y_{adj}$ is the adjusted phenotype of the m th cow, $a_{mn}$ is the n th random regression coefficient of the additive genetic effects of the m th cow, $p_{mn}$ is the *n*th random regression coefficient of the permanent environmental effect of the m th cow, $\;L_{n}\left(\omega_{t}\right)$ is the n th covariate of the Legendre polynomial at DIM_t_, $\omega_{t}$ is the normalized value of DIM_t_, and $e_{t}$ is the random residual at DIM_t_. The distribution and correlation of the adjusted phenotypes are shown in Supplementary Figure S3. The genetic variance, permanent environmental variance, heritability of traits, and genetic correlations between $t_{1}$ day in milk (DIM_t1_) and $t_{2}$ day in milk (DIM_t2_) were estimated as follows (Schaeffer et al., 2000):

$$\begin{array}{c} \sigma_{at}^{2}=L_{t}^{\prime }\hat{G}L_{t} \end{array},$$ (3)

$$\begin{array}{c} \sigma_{pet}^{2}=L_{t}^{\prime }\hat{P}L_{t} \end{array},$$ (4)

$$\begin{array}{c} \sigma_{a\left(t1,t2\right)}^{2}=L_{t}^{\prime }\hat{G}L_{t2} \end{array},$$ (5)

$$\begin{array}{c} r_{a\left(t1,t2\right)}=\frac{\sigma_{a\left(t1,t2\right)}^{2}}{\sqrt{\sigma_{a\left(t1\right)}^{2}*\sigma_{a\left(t2\right)}^{2}}} \end{array},$$ (6)

$$\begin{array}{c} h_{t}^{2}=\frac{\sigma_{at}^{2}}{\sqrt{\sigma_{at}^{2}+\sigma_{pet}^{2}+\sigma_{et}^{2}}} \end{array},$$ (7)

$$\;h_{T}^{2}=\sum_{t1=5}^{305}\sum_{t2=5}^{305}\sigma_{a\left(t1,t2\right)}^{2}/\left(\sum_{t1=5}^{305}\sum_{t2=5}^{305}\sigma_{a\left(t1,t2\right)}^{2}+\sum_{t1=5}^{305}\sum_{t2=5}^{305}\sigma_{pe\left(t1,t2\right)}^{2}+\sum_{5}^{305}\sigma_{e}^{2}\right),$$ (8)

where $\sigma_{at}^{2}$ is the genetic variance at DIM_t_, $\sigma_{pet}^{2}$ is the permanent environmental variance at DIM_t_, $\sigma_{a\left(t1,t2\right)}^{2}$ is the genetic covariance between DIM_t1_ and DIM_t2_, $L_{t}$ is a vector of Legendre polynomials at DIM_t_, $\;r_{a\left(t1,t2\right)}$ denotes the genetic correlations between DIM_t1_ and DIM_t2_, $\hat{G}$ and $\hat{P}$ are the estimated covariance matrixes for the random regression coefficients of the genetic and permanent environmental elements, respectively, $\;h_{t}^{2}$ is the heritability at DIM_t_ of the trait, $\;h_{T}^{2}$ is the heritability of the trait of the 305-day performance, and $\;\sigma_{e}^{2}$ is the residual variance.

### Principal Component Analysis

The dairy cow populations used in this study came from four different farms. To identify the population stratification, a principal component analysis (PCA) was performed on the 984 genotyped cows using the FactoMineR package in the R language (Le et al., 2008).

### Genome-Wide Association Studies

The FarmCPU (Fixed and random model Circulating Probability Unification) method, based on the multilocus linear mixed model, was used to perform the genome-wide association analysis in this study (Liu et al., 2016). The SNP genotypes coded for the association analyses were converted to 0, 1, and 2 using the Plink software v1.90 (Purcell et al., 2007). Two models were included in the FarmCPU method: a fixed effects model and a random effects model. The markers that exceeded the significant threshold in the fixed effects model were detected as pseudo quantitative trait nucleotides (QTNs). Then, the pseudo QTNs were further verified by the random effect model, where the kinships were constructed using alternative sets of pseudo QTNs (Liu et al., 2016). Iterative calculations were carried out through the fixed effects model and the random effects model, until no updated pseudo QTNs exceeded the significance threshold (Liu et al., 2016). The first five highest principal components (PCs), which explained 40% of the population stratification, were considered as covariates in the fixed effects model, in order to account for the other genetic variations except for the pseudo QTNs (Marees et al., 2018). The fixed effects model was as follows (Liu et al., 2016):

$$\begin{array}{c} y=Xb_{X}+M_{t}b_{t}+S_{j}d_{j}+e \end{array},$$ (9)

where y is the vector of the adjusted phenotypic values for MY, MFP, MFY, MPP, or MPY; $b_{X}$ is the corresponding effect of the first five PCs and **X** is the corresponding coefficient matrix; $b_{t}$ is the fixed effect of the t th pseudo QTN, which was detected by the fixed effect model and optimized by the random effect model in each cycle and $M_{t}$ is the corresponding genotype matrix; $S_{j}\;$ is the genotype of the j th marker, which was converted to 0, 1, or 2, and $d_{j}$ is the effect of the j th marker; and e is the random residual of the model. The random effect model is as follows (Liu et al., 2016):

$$\begin{array}{c} y=u+e \end{array},$$ (10)

where y is the vector of the adjusted phenotypic values of MY, MFP, MFY, MPP, or MPY;  u is the vector of total genetic effects of individuals and is assumed to satisfy ***u*** = (**0**, $K\sigma_{u}^{2}$ ), in which K is the kinship matrix constructed by the QTNs derived from the fixed effect model, and $\sigma_{u}^{2}$ is the unknown genetic variance; and e is the random residual of the model. The explained phenotypic variation (EVP) of the candidate SNPs in each trait was calculated as follows (Dadousis et al., 2017a):

$$\begin{array}{c} EVP_{n}=\frac{2*MAF_{n}*\left(1-MAF_{n}\right)*\beta_{n}^{2}}{\sigma_{y}^{2}} \end{array},$$ (11)

where $EVP_{n}$ is the explained phenotypic variation of the n th SNP, $MAF_{n}$ is the minor allele frequency of the n th SNP, $\beta_{n}\;$ is the regression coefficient of the phenotype to the  n th corresponding SNP, and $\;\sigma_{y}^{2}$ is the variance of the adjusted phenotypic values.

The Bonferroni correction method (Armstrong, 2014) was used as the threshold to verify the significance of SNPs, the type I error rate in hypothesis testing was set to 5%, and the significance threshold of the association analysis was 5.90 × 10^−7^ (0.05/84,407).

### Annotation and Enrichment Analysis of Candidate Genes

The nearest genes and the genes within 200 kilobases (kb) of the significant SNPs were selected as the candidate genes of traits, according to the bovine reference genome ARS-UCD1.2 in the UCSC database (ftp://hgdownload.soe.ucsc.edu/goldenPath/bosTau9/). To better understand the biological interactions between these candidate genes, the Kyoto Encyclopedia of Genes and Genomes (KEGG) pathways analysis and Gene Ontology (GO) analysis were conducted using the Database for Annotation, Visualization, and Integrated Discovery (DAVID) online software (https://david.ncifcrf.gov) (Dennis et al., 2003).

## Results

### Variance Components and Genetic Parameters

**FIGURE 1:**
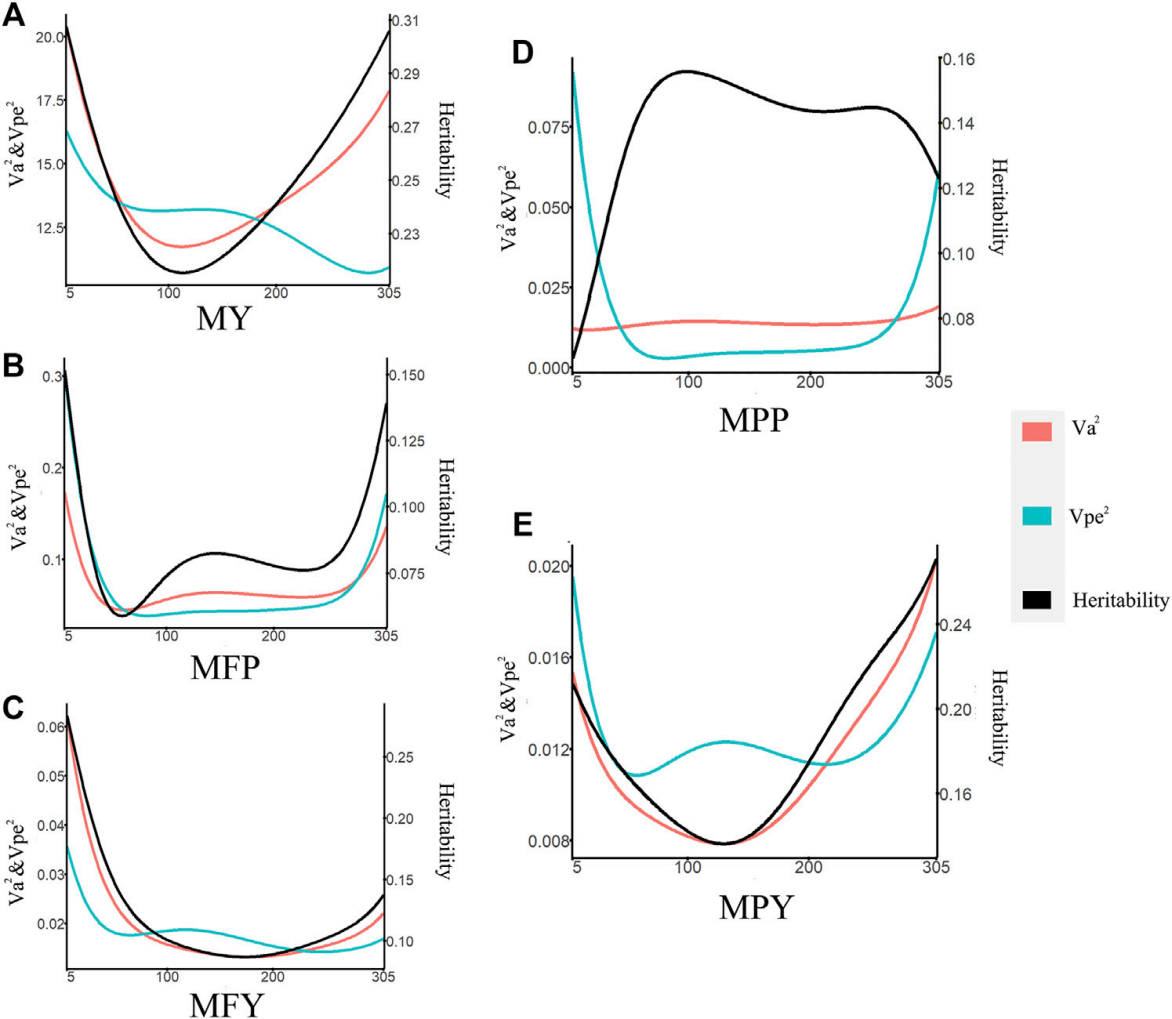
Variance component estimates of milk-related traits during the lactation (red line, additive genetic variance; blue line, permanent environmental variance; black line, heritability; **(A)** MY, milk yield; **(B)** MFP, milk fat percentage; **(C)** MFY, milk fat yield; **(D)** MPP, milk protein percentage; **(E)** MPY, milk protein yield).

The heritabilities of the 305-day MY, MFP, MFY, MPP, and MPY were 0.34, 0.29, 0.27, 0.32, and 0.28, respectively (Table 1). The variance components and heritability of these five milk-related traits at different DIMs are shown in Figure 1. In the early lactation period (5–100 days), the additive genetic variances $\left(\sigma_{a}^{2}\right)$ and the heritability of MY, MFY, and MPY all showed downward trends, reached the lowest value at the mid lactation period (100–200 days), and then increased gradually until the end of lactation (Figures 1A,C,E). The $\sigma_{a}^{2}$ and the heritability of MFP decreased and reached its lowest value at the middle of the early lactation period (5–100 days), showed an upward trend and reached a peak at the mid lactation period (100–200 days), then decreased again and reached a peak at the middle of the late lactation (200–305 days), and finally gradually increased again, until the end of the lactation (Figure 1B). The $\sigma_{a}^{2}$ of MPP was stable throughout the entire lactation (5–305 days), with a small magnitude in its changes (from 0.008 to 0.013; Figure 1D). The trend of heritability of MPP increased rapidly in the early lactation period (5–100 days), reached a maximum value at the end of the early lactation period (5–100 days), and then decreased gradually until the end of the lactation, which was approximately opposite to the trend of MFP (Figure 1B). The heritability of the 305-day performance was out of the range of heritabilities for trait performance in single test days. This could be because the heritability of the 305-day performance involves not only variance in each day but also covariance between days.

The changes in the permanent environmental variance ( $\sigma_{pe}^{2})$ of MY, MFY, and MPY were similar. All of these traits showed downward trends and reached a peak at the early lactation period (5–100 days), then increased gradually and reached a peak at the middle of the mid lactation period (100–200 days), and then decreased again gradually and reached a peak in the late lactation (200–305 days), and decreased gradually again until the end of the lactation (Figures 1A,C,E). For MFP and MPP, the changes in $\;\sigma_{pe}^{2}$ decreased rapidly and reached a minimum value for the entire lactation stage at the end of the early lactation period (5–100 days), then increased slowly until the middle of the late lactation (200–305 days), and then increased until the end of the entire lactation stage (Figures 1A,D).

### Genetic Correlations and Permanent Environmental Correlations

**FIGURE 2:**
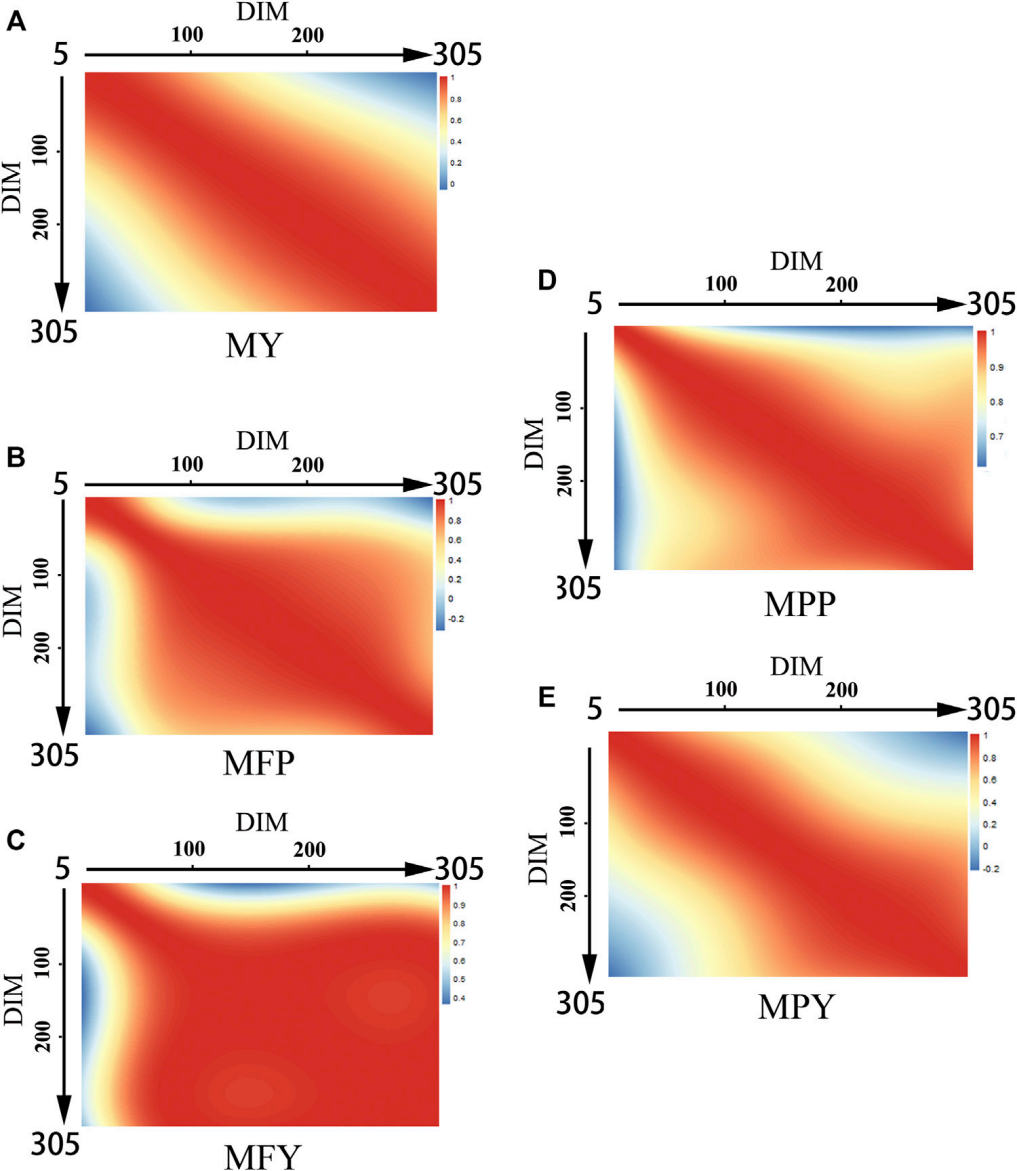
Genetic correlations of milk-related traits in different DIM during lactation (**(A)** MY, milk yield; **(B)** MFP, milk fat percentage; **(C)** MFY, milk fat yield; **(D)** MPP, milk protein percentage; **(E)** MPY, milk protein yield).

The genetic correlations between MY, MFP, MFY, MPP, and MPY and DIM are shown in Figure 2. The genetic correlations between the DIM of the five milk-related traits showed downward trends as the time interval increased, and the lowest genetic correlations were between the beginning of the early lactation (5–100 days) and the end of late lactation (200–305 days; Figure 2). The genetic correlations between the DIM for MY and MPY were higher within each lactation stage than in different lactation stages, and as the DIM interval increased, they decreased gradually (Figures 2A,E). However, for MFP, MFY, and MPP, the genetic correlations between DIM were high (>0.8) from the beginning to the middle of the early lactation period (5–100 days), and from the middle of the early lactation period (5–100 days) to the end of the entire lactation, as shown in Figures 2B–D.

The permanent environmental correlations between MY, MFP, MFY, MPP, and MPY and DIM are shown in Supplementary Figure S1. The permanent environmental correlations between DIM and MY were high (>0.8) within each lactation stage, and the correlations showed a gradually declining trend as the time interval increased (Supplementary Figure S1A). For MFY and MPY, the permanent environmental correlations between DIM were high (>0.8) from the beginning to the middle of the early lactation period (5–100 days), and from the middle of the early lactation period (5–100 days) to the end of the entire lactation stage (Supplementary Figures S1C,E). For MFP and MPP, the permanent environmental correlations between DIM were high (>0.8) from the middle of the early lactation period (5–100 days) to the middle of late lactation (200–305 days). For these two traits, although the correlations were high during the period from the beginning to the middle of early lactation, and from the middle to the end of late lactation, the correlation between early lactation and late lactation was low (<0.2), or even negative (Supplementary Figures S1B,D).

### Distributions and Correlations of Phenotypes

The distributions and the correlations of the phenotypes and the adjusted phenotypes of these milk-related traits are shown in Supplementary Figures S2, S3. The distribution of raw phenotypic values was irregular (Supplementary Figure S2), and after adjustment, the adjusted phenotypes were approximately normally distributed (Supplementary Figure S3). The correlations among the adjusted phenotypes of the five traits all significantly differed from zero (*p* < 0.05; Supplementary Figure S3). The correlations among the adjusted values of MY, MFY, and MPY were high (>0.78), and even reached 0.91 between MY and MPY. The correlations between the adjusted values of MY and MFP, and MY and MPP were significantly negative (Supplementary Figure S3). The correlations between the adjusted values of MFP and MFY were significantly positive (0.28), contrary to the correlations between MPP and MPY (−0.25; Supplementary Figure S3).

### Marker Information and Population Structure

**FIGURE 3:**
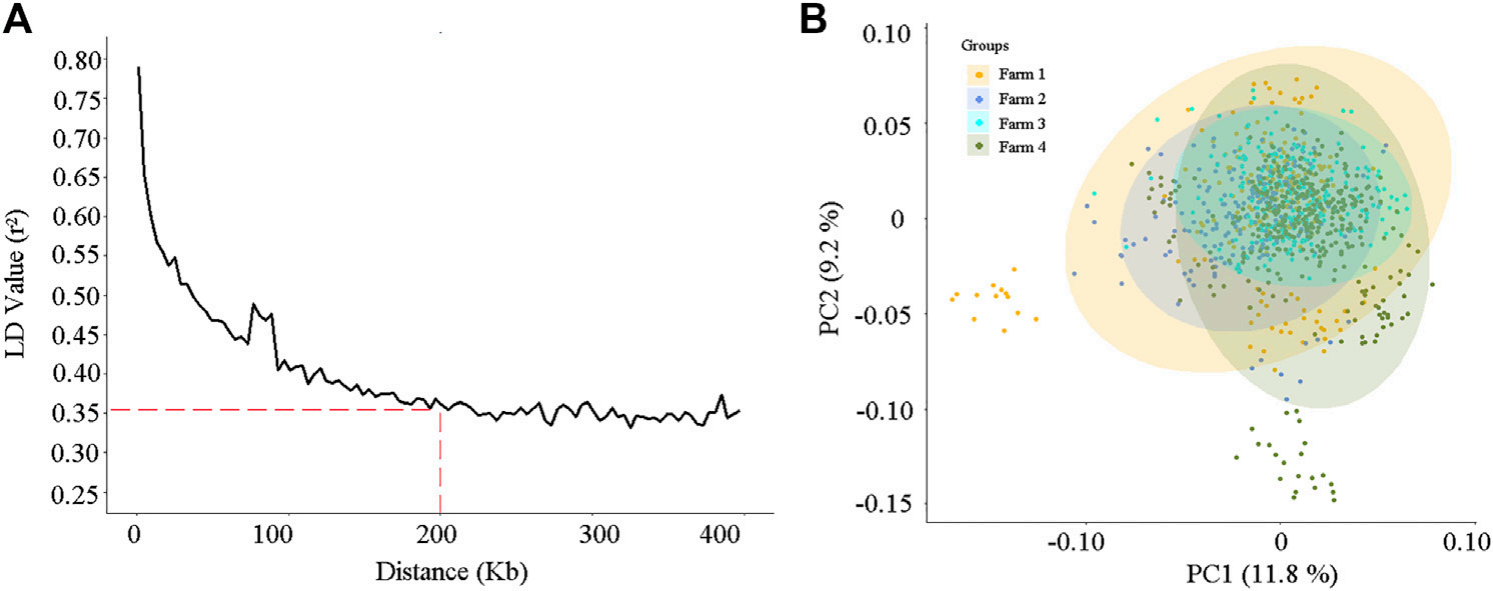
The linkage disequilibrium decay analysis **(A)** and the principal component analysis **(B)** according to the 84,407 single nucleotide polymorphisms (SNPs) for the 984 cows.

After quality control, a total of 84,407 variants on 30 chromosomes remained for association analysis. The relationship between the linkage disequilibrium (LD) value (r^2^) and the average distance of markers is shown in Figure 3A. As the distance increased, the average LD value between markers decreased, and the average r^2^ exceeded 0.35 when the distance of markers was less than 200 kb (Figure 3A).

Principal component analysis (PCA) was conducted on the 984 cows, according to the variant information. As shown in Figure 3B, stratification existed in our study population, and some cows in farms 1 and 4 were separated from other individuals. The first two highest PCs explained 11.8% and 9.2% of the variation, respectively (Figure 3B). To reduce the spurious genetic associations generated by population stratification, the first five PCs, which explained 40% of the population variation, were considered and incorporated into the fixed effect model of GWAS.

### Genome-Wide Association Study

**FIGURE 4:**
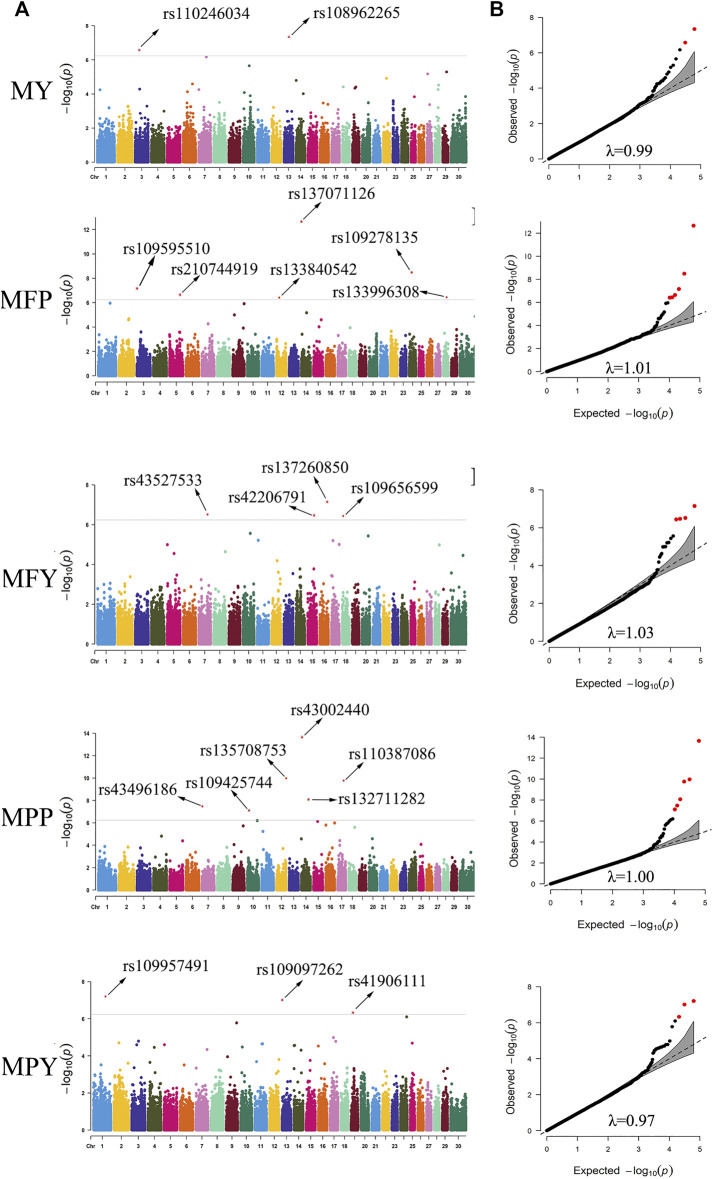
Manhattan plots **(A)** and quantile-quantile (QQ) plots **(B)** demonstrated from the genome-wide association study (GWAS) results of the milk-related traits. The significance threshold was 5.90 × 10^−7^. The red dots represent the significant SNPs. The abscissas and ordinates in the Manhattan plots represent the 30 chromosomes of cows and the negative logarithms of the *p*-values of the variants, respectively **(A)**. The abscissas and ordinates in the QQ plots represent negative logarithms of the expected *p*-values and negative logarithms of the observed *p*-values of each SNP **(B)** (MY. milk yield; MFP. milk fat percentage; MFY. milk fat yield; MPP. milk protein percentage; MPY. milk protein yield, λ. inflation factor value).

In this study, 21 SNPs passed the Bonferroni correction threshold (0.05/84,407) and were significantly associated with the five milk-related traits (Figure 4A). Two SNPs (rs108962265 and rs110246034), located on chromosomes 13 and 3, were significantly associated with the trait MY, and the nearest genes to them were *PITRM1* (194 kb) and *PRMT6* (788 kb), respectively. Six SNPs (rs137071126, rs109278135, rs109595510, rs210744919, rs133996308, and rs133840542), located on chromosomes 14, 24, 3, 5, 28, and 12, were significantly associated with the trait MFP, with the nearest genes to them being *SLC52A2* (within), *NOL4* (139 kb), *RCSD1* (within), *MGST1* (within), *PLAU* (1 kb), and *SUPT20H* (within), respectively. Four SNPs (rs137260850, rs43527533, rs42206791, and rs109656599), located on chromosomes 16, 7, 15, and 18, were significantly associated with the trait MFY, and the nearest genes to them were *PLA2G4A* (75 kb), *TENM2* (within), *METTL15* (314 kb), and *CDH13* (within), respectively. Six SNPs (rs43002440, rs135708753, rs110387086, rs132711282, rs43496186, and rs109425744), located on chromosomes 14, 12, 17, 14, 7, and 10, were significantly associated with the trait MPP, and the nearest genes to them were *KHDRBS3* (31 kb), *ATP11A* (within), *MLXIP* (within), *FBXO43* (within), *WNT9A* (44 kb), and *CORO2B* (37 kb), respectively. Three SNPs (rs109957491, rs109097262, and rs41906111), located on chromosomes 1, 13, and 19, were significantly associated with the trait MPY, the nearest genes to them being *MFSD1* (40 kb), *PLCB1* (288 kb), and *MYO1D* (8 kb), respectively. The detected significant SNPs explained 5.44%, 12.39%, 8.89%, 10.65%, and 7.09% of the phenotypic variation of MY, MFP, MFY, MPP, and MPY, respectively. The summary statistics of these 21 SNPs are presented in Table 2.

**TABLE 2:** Information of the identified 21 variants in the association analysis.

Trait	SNP	CHR	Position	Nearest gene	Distance (kb)	MAF	EVP	*p* value
MY	rs108962265	13	44,887,254	*PITRM1*	−193.90	0.0753106	3.24%	4.53 × 10^−8^
	rs110246034	3	37,101,102	*PRMT6*	+788.23	0.380435	2.20%	2.64 × 10^−7^
MFP	rs137071126	14	580,019	*SLC52A2*	Within (extro)	0.244565	3.46%	2.20 × 10^−13^
	rs109278135	24	22,711,564	*NOL4*	−138.54	0.437888	2.50%	3.26 × 10^−9^
	rs109595510	3	1,012,940	*RCSD1*	Within (exon)	0.462733	2.29%	6.76 × 10^−8^
	rs210744919	5	93,520,138	*MGST1*	Within (intron)	0.298137	1.33%	2.23 × 10^−7^
	rs133996308	28	29,790,048	*PLAU*	−1.06	0.306677	1.32%	3.59 × 10^−7^
	rs133840542	12	24,656,370	*SUPT20H*	Within (intron)	0.295031	1.49%	3.84 × 10^−7^
MFY	rs137260850	16	68,156,133	*PLA2G4A*	+74.85	0.435559	2.73%	7.08 × 10^−8^
	rs43527533	7	80,246,600	*TENM2*	Within (intron)	0.384317	1.99%	3.03 × 10^−7^
	rs42206791	15	59,334,965	*METTL15*	+313.75	0.166149	2.00%	3.40 × 10^−7^
	rs109656599	18	9,410,999	*CDH13*	Within (intron)	0.152174	2.17%	3.66 × 10^−7^
MPP	rs43002440	14	6,369,558	*KHDRBS3*	−31.17	0.338509	3.80%	2.22 × 10^−14^
	rs135708753	12	86,298,432	*ATP11A*	Within (intron)	0.481366	2.02%	1.04 × 10^−10^
	rs110387086	17	53,247,625	*MLXIP*	Within (intron)	0.487578	1.58%	1.67 × 10^−10^
	rs132711282	14	64,235,532	*FBX O 43*	Within (intron)	0.404503	1.16%	8.26 × 10^−9^
	rs43496186	7	3,207,173	*WNT9A*	+43.93	0.488354	1.07%	3.29 × 10^−8^
	rs109425744	10	15,382,216	*CORO2B*	−37.09	0.453416	1.02%	7.72 × 10^−8^
MPY	rs109957491	1	108,667,188	*MFSD1*	−39.24	0.190994	2.87%	6.20 × 10^−8^
	rs109097262	13	2,016,597	*PLCB4*	−287.69	0.459627	2.29%	9.60 × 10^−8^
	rs41906111	19	17,322,522	*MYO1D*	−8.28	0.476708	1.93%	4.66 × 10^−7^

Note. CHR, chromosome; MY, milk yield; MFP, milk fat percentage; MFY, milk fat yield; MPP, milk protein percentage; MPY, milk protein yield; MAF, minor allele frequency; EVP, explained phenotypic variation. Distance (define within too), the negative sign indicates that the SNP is in the upstream of the gene, and the positive sign indicates that the SNP is in the downstream of the gene.

To evaluate the statistical validity and rationality of the association analysis, quantile–quantile (QQ) plots of the five milk-related traits were generated (Figure 4B). The vast majority of the SNPs conformed to the expected *p*-values, and the inflation factor (λ) values of the five milk-related traits were all close to 1 (Figure 4B), which illustrated that false positive and false negative results were well avoided in the process of association analysis. The QQ plots, as well as Manhattan plots, of the five milk-related traits are shown in Figure 4.

### Enrichment Analysis

**FIGURE 5:**
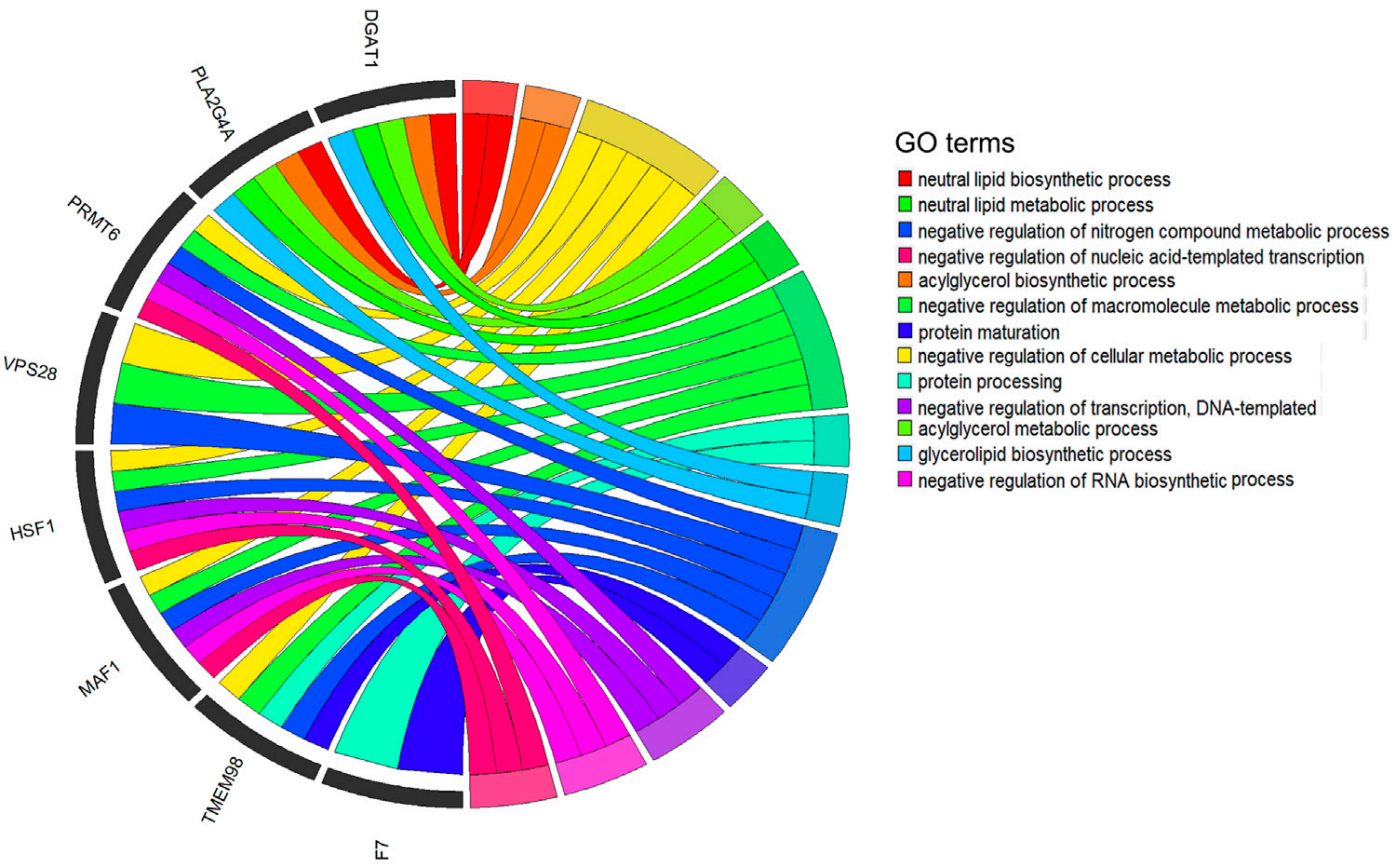
Significant Gene Ontology (GO) terms enriched by the genes within 200 kb of significant SNPs. After quality control, 13 GO terms that are enriched by eight genes are revealed in the circus plots (*p* < 0.05).

To deeply understand the biological functions of the 21 significant SNPs related to the five milk-related traits of cows (MY, MFP, MFY, MPP, and MPY), KEGG and GO analyses were conducted on the nearest genes and genes within 200 kb of these SNPs. In total, 101 genes were obtained (Supplementary Table S1). These genes were significantly enriched in metabolism and decomposition of amino acids pathways and nerve signal transduction pathways (Table 3, *p* < 0.05), such as melanogenesis, Wnt signaling pathway, GnRH signaling pathway, circadian entrainment, signaling pathways regulating the pluripotency of stem cells, phospholipase D signaling pathway, oxytocin signaling pathway, glutathione metabolism, mTOR signaling pathway, and long-term potentiation (Table 3). Twenty-five GO terms were significantly enriched (Supplementary Table S2, *p* < 0.05). After removing the terms that contained only one gene and the genes that were enriched in only one term, 13 GO terms enriched by eight genes were retained (Figure 5), which mainly participated in the progress of metabolism of fat and protein, as well as regulation of transcription, such as neutral lipid biosynthetic process, acylglycerol biosynthetic process, negative regulation of the cellular metabolic process, acylglycerol metabolic process, neutral lipid metabolic process, negative regulation of macromolecule metabolic process, protein processing, glycerolipid biosynthetic process, negative regulation of nitrogen compound metabolic process, protein maturation, negative regulation of transcription, DNA-templated negative regulation of RNA biosynthetic process, and negative regulation of nucleic acid-templated transcription. Eight of the 101 genes were detected in the above 13 biological processes (Figure 5).

**TABLE 3:** Details of the Kyoto Encyclopedia of Genes and Genomes (KEGG) pathways significantly enriched from the nearest genes and the genes within 200 kb of the significant single-nucleotide polymorphisms (SNPs).

Pathway	Description	Gene name	*p*-Value
bta04916	Melanogenesis	*CAMK2G*, *WNT3A*, *WNT9A*, *PLCB4*	0.001
bta04310	Wnt signaling pathway	*CAMK2G*, *WNT3A*, *WNT9A*, *PLCB4*	0.006
bta04912	GnRH signaling pathway	*CAMK2G*, *PLA2G4A*, *PLCB4*	0.008
bta04713	Circadian entrainment	*ADCY10*, *CAMK2G*, *PLCB4*	0.010
bta04550	Signaling pathways regulating pluripotency of stem cells	*SMAD9*, *WNT3A*, *WNT9A*	0.026
bta04072	Phospholipase D signaling pathway	*PLA2G4A*, *ARF1*, *PLCB4*	0.030
bta04921	Oxytocin signaling pathway	*CAMK2G*, *PLA2G4A*, *PLCB4*	0.030
bta00480	Glutathione metabolism	*OPLAH*, *MGST1*	0.031
bta04150	mTOR signaling pathway	*CLIP1*, *WNT3A*, *WNT9A*	0.032
bta04720	Long-term potentiation	*CAMK2G*, *PLCB4*	0.038

## Discussion

A test-day model (TDM) can account for the persistence and the test-day effect on individual phenotypes, making it a powerful method to improve the reliability of EBV estimation (Schaeffer et al., 2000). The additive genetic variances, permanent environmental variances, and the heritabilities of five milk-related traits changing during lactation were assessed in this study (Figure 1), which revealed that the genes or the expression of genes that control these milk-related traits might differ along the lactation trajectory of cows (Wahinya et al., 2020). The scope of the additive genetic variances of the MY in this study (Figure 1A) was higher than that in cows in North Carolina and Portugal (Silvestre et al., 2005), but was in the same range as the cows in medium- and high-production systems in Kenya (Wahinya et al., 2020). Furthermore, the trend and range of additive genetic variations of MY during lactation (Figure 1A) were consistent with those of Japanese Holstein cows (Togashi et al., 2008). Many studies have shown that the cows with high MY have greater genetic variances than low-yield cows (Togashi et al., 2008; Wahinya et al., 2020). These higher additive variances indicate that the cows are more genetically diverse (Wahinya et al., 2020). The genetic variances and heritability at the DIM_5_–DIM_50_ and DIM_250_–DIM_305_, with respect to the MY, were higher than in other periods in this study, indicating that genetic improvement for performance during these two periods by selection could be more efficient than when considering other periods (Figure 1A).

The trajectories of additive genetic variances and the heritability of MFY and MPY with DIM (Figures 1C,E) were the same in the studies performed on cows in the United States; that is, they decreased in early lactation and then increased until the end of the lactation, while in the middle lactation stage, they all reached the minimum values over the whole lactation (DeGroot et al., 2007). Therefore, in the mid lactation period, the genetic improvement space for MFY and MPY of dairy cows was smaller than that in the early and late lactation periods. The scope and trend of the heritability of MFY (Figure 1C) were also the same as those for Tunisian Holsteins, but the trend for MPY was different (Figure 1E) (Hammami et al., 2008), which may have been due to the permanent environmental variances during the mid lactation period of the Tunisian Holstein population in the study of Hammami, being less than that observed in the cows in our research. The changes in the genetic variances and the heritability with DIM of MFP were similar to those of MFY, but the lowest values of the genetic variances and the heritability of MFP appeared in the middle of the early lactation period, earlier than for MFY (Figures 1B, C). The genetic variances of MPP were very stable over the whole lactation, but the permanent environmental variances at the beginning and the end of lactation changed dramatically, which resulted in the heritability of MPP reaching the lowest value at the beginning of early lactation (Figure 1D). This may be due to the specific environmental influences in the different farms, such as calving preparation and dry cow management (Gengler and Wiggans, 2001; Hammami et al., 2008). Our study also found that heritability of milk fat traits (MFY and MFR) was, most of the time during lactation (89.70% and 91.69%, respectively), lower than that for the corresponding milk protein traits (MPY and MPP; Figure 1), which was in agreement with previous studies (Tijani et al., 1999; Silvestre et al., 2005).

The genetic correlations between the DIM for the five milk-related traits were high through the adjacent days, and then decreased gradually as the time interval increased (Figure 2), similar to the results of the group of Wahinya (Wahinya et al., 2020). The genetic correlations between the beginning of the early lactation period and the end of the late lactation period were not high (<0.4), sometimes even being negative (MFP and MPY; Figures 2B,E). This indicated that it might not be reasonable to predict the milk potential of cows according to only their performance during the early lactation period (Bignardi et al., 2009b; Ojango et al., 2019). Therefore, keeping a record along the whole lactation is necessary for a reliable genetic evaluation of dairy cows in the lower reaches of the Yangtze River, especially for MY and MPY, due to the genetic correlation between DIM during different lactation stages being lower than that within each lactation stage (Figures 2A,E). For MFP, MFY, and MPP, the genetic correlations from DIM_50_ to DIM_305_ were as high than 0.8, and extremely few individual records missing in the middle and late stages of lactation might not make an excessive impact on the accuracy of the overall genetic evaluation (Figures 2B–D).

Analyses of linkage disequilibrium (LD) and population stratification are the premises and important steps for association studies (Bulik-Sullivan et al., 2015; Lu et al., 2021). In this study, the degree of LD (r^2^) showed a downward trend as the average distance between SNPs increased (Figure 3A). It is worth noting that in beef cattle populations, such as Angus cattle, Brahman cattle, Belmont Red cattle, Santa Gertrudis cattle, and Iranian water buffalo (Porto-Neto et al., 2014; Mokhber et al., 2019), the decay rate is much higher than that observed for the dairy cows in this research. This might be due to the process of artificial domestication and selection in beef cattle being slower than that for dairy cows (Porto-Neto et al., 2014). In the present study, the r^2^ of the LD was approximately equal to 0.35 when the average distance of SNPs was 200 kb, and it increased as the average SNPs distance decreased (Figure 3A). Two hundred kilobases is a common distance, which has been used to find and annotate genes related to SNPs in previous association studies (Sanchez et al., 2017; Mota et al., 2020; Lu et al., 2021). Selecting the most significant principal components (PCs), which were constructed from the SNP variables of the research population, as the covariates in the fixed effect model of the GWAS provides an effective method to reduce the effects produced by population stratification (Marees et al., 2018; Jiang et al., 2019b). In the present study, the PCA scatter plot showed that population stratification, indeed, appeared in our study population, and the highest two PCs explained 21% of the variation (Figure 3B). After proper correction, the inflation factor (λ) values of the five milk-related traits were all close to 1 (Figure 4B), and the overwhelming majority of observed *p*-values of the SNPs were in line with our expectations (Figure 4B), indicating that the population stratification was effectively in control (Price et al., 2010).

In this study, a total of 21 SNPs exceeding the significance threshold (5.90 × 10^−7^) were identified to be associated with MY, MFP, MFY, MPP, and MPY (Figure 4A). Among these SNPs, some were in QTL regions that have been reported previously, such as rs137071126 (Buitenhuis et al., 2014; Fang et al., 2014), rs42206791 (Jiang J. et al., 2019), rs43002440 (Jiang J. et al., 2019), rs132711282 (Jiang J. et al., 2019), and rs109097262 (Cole et al., 2011). The genes nearest these 21 SNPs are shown in Table 2, some of which have been confirmed to be related to the corresponding milk-related traits. *PITRM1* has been reported to be related to the milk production progress in Reggiana cows (Bertolini et al., 2020). *SLC52A2* plays a potential role in the mammary gland and affects the fatty acid content in dairy cows (Palombo et al., 2018). *MGST1* has been identified to be highly associated with MFP and other milk composition traits in cattle (Littlejohn et al., 2016). It was reported that the expression level of *PLAU* activity is high in the peak lactation periods of dairy cows and could affect the composition of milk fatty acids (Wickramasinghe et al., 2012). *PLA2G4A* participates in catalyzing the hydrolysis of membrane glycerophospholipids, promoting the production of free fatty acids in milk (Diouf et al., 2006). *CDH13* has been reported to be associated with milk cholesterol in cows (Do et al., 2018). *FBXO43* has been primarily implicated in the MPY of cows (Tribout et al., 2020). *PLCB4* is a significant gene included in the phosphatidylinositol pathway and may affect the stability of the cream emulsion in milk (Dadousis et al., 2017b).

To further reveal the functional orientation of the significant SNPs, an enrichment analysis was performed for the genes nearest or within 200 kb of these variants, and 101 genes were found (Supplementary Table S1). Due to the high phenotypic (Supplementary Figures S2, S3) and genetic correlations (Liu et al., 2020) of these five milk-related traits, we conducted the enrichment analysis for all the candidate genes of the five traits, and 10 KEGG pathways were significantly enriched (*p* < 0.05, Table 3). All of these pathways have been reported to be highly related to milk-related traits in cattle. The melanogenesis pathway has been significantly related to the MY of river buffaloes (Menon et al., 2016). The Wnt signaling pathway has been found to be essential for the development of the mammary gland during lactation in Holstein cows (Wang et al., 2013). GnRH is synthesized and released in the hypothalamus from the GnRH neurons, and GnRH and oxytocin are strongly related to reproduction in mammals (Schneider et al., 2006); Furthermore, a close relationship has been revealed between reproduction and milk production in dairy cows (Dadousis et al., 2017a). Phospholipase D, from the phospholipase D signaling pathway, is not only the key enzyme for the generation of phosphatidic acid but also the main molecular substance in the synthesis of milk fat. In Thai multibreed dairy population, the Wnt signaling pathway, the phospholipase D signaling pathway, and circadian entrainment could explain 0.31, 0.47, and 0.38% of the genetic variance in MY, respectively, and explained 0.30, 0.44, and 0.36% of the genetic variance in MFY, respectively (Laodim et al., 2019). Folic acid regulates many key metabolic-related genes in the glutathione metabolism pathway, and the secretion of folic acid may increase milk yield in the perinatal period of dairy cows (Khan et al., 2020). Melatonin can inhibit the mTOR signaling pathway and suppress milk fat synthesis in bovine mammary epithelial cells (Wang Y. et al., 2019). Long-term potentiation has been related to the amino acid composition of the MFP, which could affect the taste and flavor of milk (Dadousis et al., 2017a). We speculate that genes in these pathways could be candidate genes for milk-related traits in cows.

The genes nearest or within 200 kb of the significant SNPs were significantly enriched in 25 GO terms (*p* < 0.05, Supplementary Table S2). To discover the core terms and genes affecting the milk performance of cows, the GO terms that contained only one gene and the genes enriched in only one term were removed. Finally, 13 GO terms enriched by eight genes remained for analysis (Figure 5). These 13 terms were mainly involved in the lipid biosynthetic and metabolic process, protein maturation process, regulation of transcription process, and regulation of macromolecule metabolic processes (Figure 5), which are all highly related to milk-related traits of cows (Bauman et al., 2006; Osorio et al., 2016). Furthermore, eight of the candidate genes were detected in these processes frequently (Figure 5). It is well known that the *DGAT1* gene is a fundamental metabolic enzyme that plays important roles in triglyceride biosynthesis, glyceride metabolism, and the digestion and absorption of the fat in milk (Yen et al., 2008). Many previous studies have revealed the relationships between *DGAT1* and milk-related traits, especially that the polymorphism of K232A in *DGAT1* significantly influences the MFY and MPY (Bovenhuis et al., 2016; Yao et al., 2021). *VPS28* may regulate milk fat synthesis through ubiquitylation in bovine mammary epithelial cells (Liu and Zhang, 2020). It has been reported that a single nucleotide polymorphism of *HSF1* is related to the milk fatty acid composition in Italian Holstein cows (Palombo et al., 2018). *MAF1* is a candidate gene related to MY and MPY in Canadian Holstein cows (Oliveira et al., 2018). One nonsynonymous coding SNP (rs136905662, p.Gly265Val, c.794G>T) on the *F7* gene has been reported to be associated with the content of caproic acid in milk (Ibeagha-Awemu et al., 2016). Therefore, we speculated that these genes may play key roles in the variation of these milk-related traits.

## Conclusion

In this study, we estimated the genetic parameters of five milk-related traits using a random regression test-day model, and performed genome-wide association analyses on these traits. The additive genetic variances, the permanent environmental variances, and the heritabilities of these traits constantly changed throughout the whole lactation, and the genetic correlations and permanent environmental correlations between different DIMs within lactation decreased as the time interval increased. A total of 21 SNPs were detected as being significantly associated with these milk-related traits, five of which were located in QTL regions that have been previously reported. We also found 17 candidate genes that may play key roles in the phenotypic variation of these traits. The presented results could be useful for understanding the basis of quantitative genetics and the genetic architecture of milk-related traits in dairy cows, thus, contributing to the genetic improvement of dairy cows in the lower reaches of the Yangtze River.

## Data Availability

The original contributions presented in the study are included in the article/Supplementary Material, further inquiries can be directed to the corresponding authors.
