# Supplementary material for: Genetic Parameter Estimation and Genome-Wide Association Study-Based Loci Identification of Milk-Related Traits in Chinese Holstein
Source: Front Genet. 2022 Jan 28;12:799664. doi: 10.3389/fgene.2021.799664 (PMC8836289; doi:10.3389/fgene.2021.799664)
Supplement: Supplementary file 1 [file DataSheet1.ZIP › Supplementary_Material.docx]

Supplementary Material

# Supplementary Figures and Tables

## Supplementary Figures

**
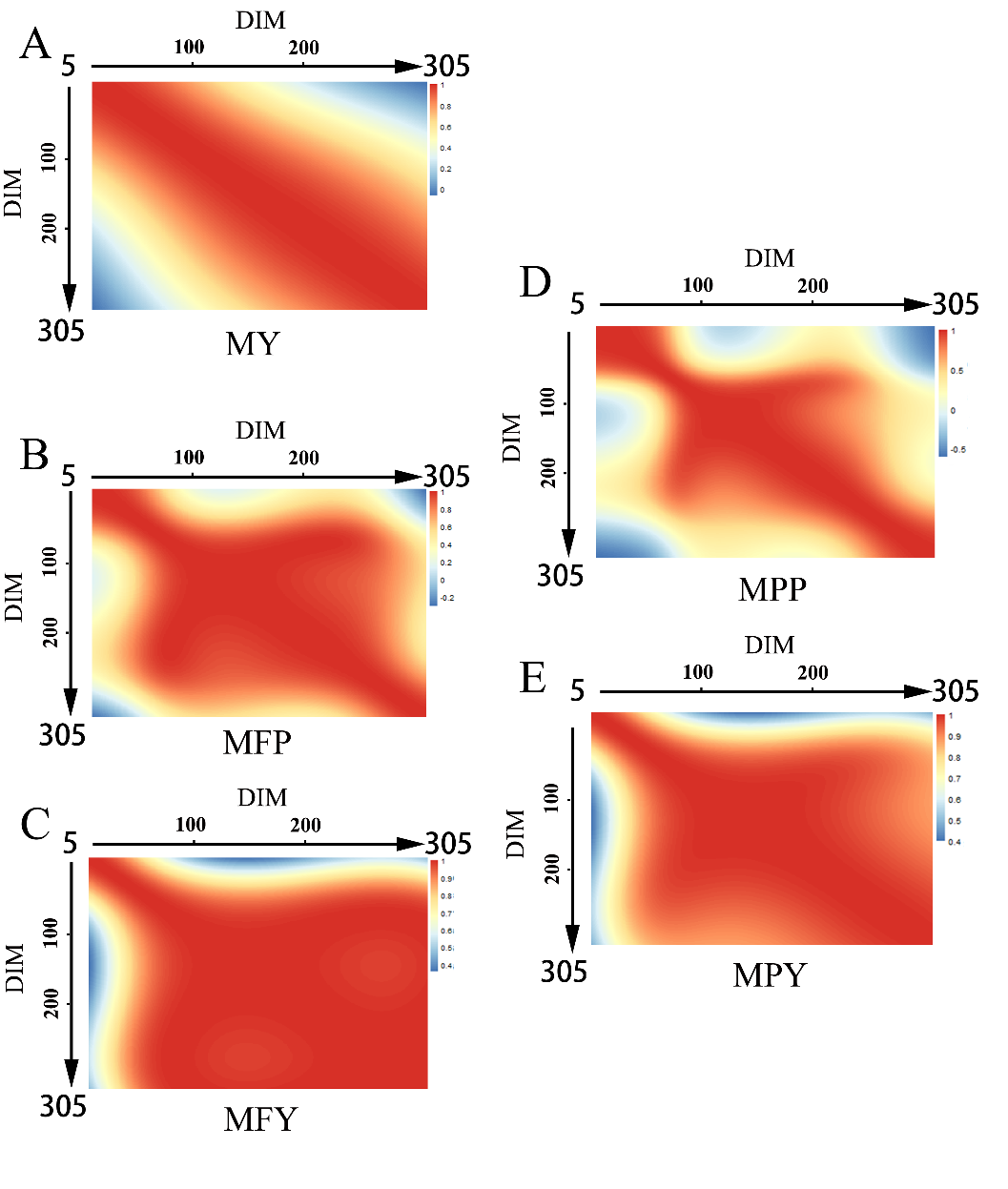
**

**Supplementary Figure 1.** Permanent environmental correlations of milk-related traits in different DIM during lactation. (MY: milk yield; MFP: milk fat percentage; MFY: milk fat yield; MPP: milk protein percentage; MPY: milk protein yield).

**
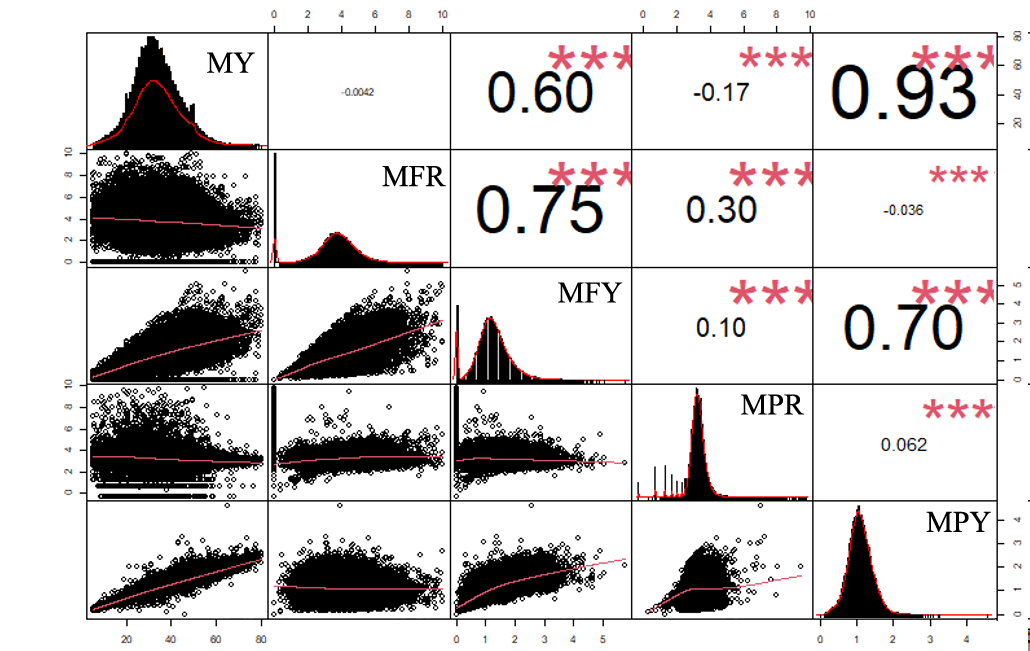
**

**Supplementary Figure 2.** The distributions and the correlations of the raw phenotypes. (MY: milk yield; MFP: milk fat percentage; MFY: milk fat yield; MPP: milk protein percentage; MPY: milk protein yield).

**
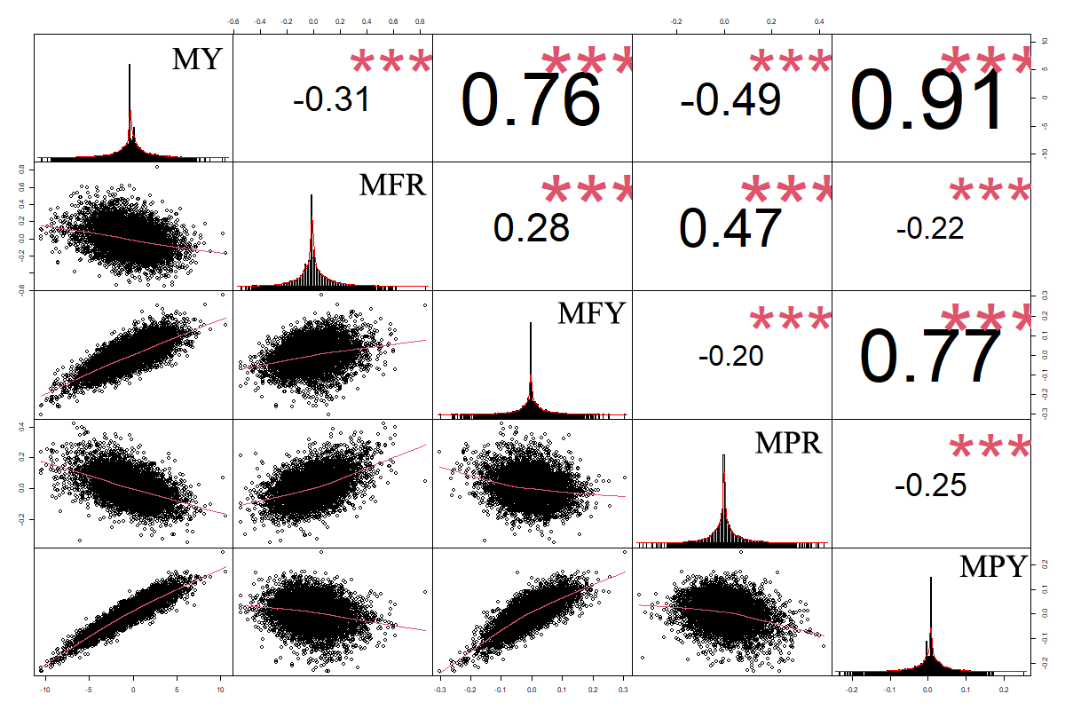
**

**Supplementary Figure 3.** The distributions and the correlations of the adjusted phenotypes. (MY: milk yield; MFP: milk fat percentage; MFY: milk fat yield; MPP: milk protein percentage; MPY: milk protein yield).

## Supplementary Table

**Table S1.** Genes within 200 kb detected from the significant SNPs of milk-related traits

| **Traits** | **SNP** | **Genes** | **Ensemble ID** | **CHR** | **Location** | **Distance (bp)** |
| --- | --- | --- | --- | --- | --- | --- |
| MY | rs108962265 | *PITRM1* | ENSBTAG00000002685 | 13 | 45107041-45161102 | -193897 |
| MFP | rs137071126 | *bta-mir-2308* | ENSBTAG00000045478 | 14 | 381366-381434 | 198585 |
|  |  | *C14H8orf82* | ENSBTAG00000046031 | 14 | 416888-419537 | 160482 |
|  |  | *LRRC24* | ENSBTAG00000004970 | 14 | 417616-424777 | 155242 |
|  |  | *LRRC14* | ENSBTAG00000004969 | 14 | 424798-428272 | 151747 |
|  |  | *RECQL4* | ENSBTAG00000010276 | 14 | 428460-434942 | 145077 |
|  |  | *MFSD3* | ENSBTAG00000007838 | 14 | 435003-437076 | 142943 |
|  |  | *PPP1R16A* | ENSBTAG00000007834 | 14 | 443246-448420 | 131599 |
|  |  | *FOXH1* | ENSBTAG00000004761 | 14 | 469132-470687 | 109332 |
|  |  | *KIFC2* | ENSBTAG00000007753 | 14 | 470777-478486 | 101533 |
|  |  | *CYHR1* | ENSBTAG00000035254 | 14 | 478582-492655 | 87364 |
|  |  | *VPS28* | ENSBTAG00000026320 | 14 | 508418-512819 | 67200 |
|  |  | *SLC39A4* | ENSBTAG00000046026 | 14 | 533918-538406 | 41613 |
|  |  | *CPSF1* | ENSBTAG00000008355 | 14 | 542386-556837 | 23182 |
|  |  | *ADCK5* | ENSBTAG00000011064 | 14 | 556020-570498 | 9521 |
|  |  | *SLC52A2* | ENSBTAG00000000857 | 14 | 578057-580805 | within |
|  |  | *FBXL6* | ENSBTAG00000000856 | 14 | 580951-583957 | -932 |
|  |  | *SCRT1* | ENSBTAG00000050112 | 14 | 594540-600190 | -14521 |
|  |  | *DGAT1* | ENSBTAG00000026356 | 14 | 603813-612791 | -23794 |
|  |  | *HSF1* | ENSBTAG00000020751 | 14 | 613328-634349 | -33309 |
|  |  | *BOP1* | ENSBTAG00000009811 | 14 | 634453-652447 | -54434 |
|  |  | *SCX* | ENSBTAG00000009816 | 14 | 646600-648020 | -66581 |
|  |  | *MROH1* | ENSBTAG00000014458 | 14 | 652785-702430 | -72766 |
|  |  | *bta-mir-1839* | ENSBTAG00000054104 | 14 | 691785-691856 | -111766 |
|  |  | *HGH1* | ENSBTAG00000039705 | 14 | 712202-715270 | -132183 |
|  |  | *WDR97* | ENSBTAG00000000658 | 14 | 720987-723829 | -140968 |
|  |  | *MAF1* | ENSBTAG00000012242 | 14 | 728324-732756 | -148305 |
|  |  | *SHARPIN* | ENSBTAG00000012235 | 14 | 732964-737292 | -152945 |
|  |  | *CYC1* | ENSBTAG00000012232 | 14 | 738124-740518 | -158105 |
|  |  | *GPAA1* | ENSBTAG00000014610 | 14 | 750608-753850 | -170589 |
|  |  | *EXOSC4* | ENSBTAG00000014607 | 14 | 755134-757010 | -175115 |
|  |  | *OPLAH* | ENSBTAG00000017281 | 14 | 765421-774581 | -185402 |
|  |  | *SPATC1* | ENSBTAG00000026350 | 14 | 778853-806391 | -198834 |
|  | rs109278135 | *NOL4* | ENSBTAG00000010299 | 24 | 22850105-23337742 | -138541 |
|  | rs109595510 | *ADCY10* | ENSBTAG00000001052 | 3 | 809356-843304 | 169636 |
|  |  | *MPZL1* | ENSBTAG00000002823 | 3 | 915375-997233 | 15707 |
|  |  | *RCSD1* | ENSBTAG00000014151 | 3 | 1011191-1083046 | within |
|  |  | *CREG1* | ENSBTAG00000008931 | 3 | 1148816-1160249 | -135876 |
|  |  | *CD247* | ENSBTAG00000012700 | 3 | 1182748-1266862 | -169808 |
|  | rs210744919 | *LMO3* | ENSBTAG00000013541 | 5 | 93264607-93328292 | 191846 |
|  |  | *MGST1* | ENSBTAG00000008541 | 5 | 93497064-93521047 | within |
|  |  | *SLC15A5* | ENSBTAG00000009444 | 5 | 93602194-93699192 | -82056 |
|  | rs133996308 | *MYOZ1* | ENSBTAG00000033008 | 28 | 29615073-29627462 | 162586 |
|  |  | *SYNPO2L* | ENSBTAG00000021132 | 28 | 29626166-29634566 | 155482 |
|  |  | *SEC24C* | ENSBTAG00000002791 | 28 | 29639357-29662099 | 127949 |
|  |  | *FUT11* | ENSBTAG00000002792 | 28 | 29662120-29665188 | 124860 |
|  |  | *CHCHD1* | ENSBTAG00000012654 | 28 | 29679227-29680517 | 109531 |
|  |  | *ZSWIM8* | ENSBTAG00000012657 | 28 | 29682252-29697213 | 92835 |
|  |  | *NDST2* | ENSBTAG00000012664 | 28 | 29696608-29705236 | 84812 |
|  |  | *CAMK2G* | ENSBTAG00000012667 | 28 | 29705563-29761013 | 29035 |
|  |  | *PLAU* | ENSBTAG00000005947 | 28 | 29791112-29799836 | -1064 |
|  |  | *VCL* | ENSBTAG00000021879 | 28 | 29876700-29989469 | -86652 |
|  | rs133840542 | *SUPT20H* | ENSBTAG00000011860 | 12 | 24646355-24685566 | within |
|  |  | *EXOSC8* | ENSBTAG00000011855 | 12 | 24685659-24694910 | -29289 |
|  |  | *ALG5* | ENSBTAG00000011850 | 12 | 24694524-24722674 | -38154 |
|  |  | *SMAD9* | ENSBTAG00000007589 | 12 | 24731844-24795963 | -75474 |
|  |  | *RFXAP* | ENSBTAG00000005351 | 12 | 24807037-24815859 | -150667 |
| MFY | rs137260850 | *PLA2G4A* | ENSBTAG00000013298 | 16 | 67906979-68081283 | 74850 |
|  | rs43527533 | *TENM2* | ENSBTAG00000025071 | 7 | 79741698-80302099 | within |
|  |  | *WWC1* | ENSBTAG00000013880 | 7 | 80336697-80493580 | -90097 |
|  |  | *bta-mir-2462* | ENSBTAG00000045238 | 7 | 80356568-80356636 | -109968 |
|  | rs109656599 | *CDH13* | ENSBTAG00000034373 | 18 | 9350020-10154230 | within |
| MPP | rs43002440 | *KHDRBS3* | ENSBTAG00000002181 | 14 | 6400724-6544685 | -31166 |
|  | rs135708753 | *SPACA7* | ENSBTAG00000054563 | 12 | 86167315-86183236 | 115196 |
|  |  | *TUBGCP3* | ENSBTAG00000044184 | 12 | 86186156-86222088 | 76344 |
|  |  | *ATP11A* | ENSBTAG00000000446 | 12 | 86288141-86389324 | within |
|  |  | *MCF2L* | ENSBTAG00000017289 | 12 | 86422451-86489884 | -124019 |
|  |  | *F7* | ENSBTAG00000007411 | 12 | 86491628-86497941 | -193196 |
|  | rs110387086 | *CLIP1* | ENSBTAG00000016779 | 17 | 52998851-53115871 | 131754 |
|  |  | *VPS33A* | ENSBTAG00000004203 | 17 | 53120968-53150578 | 97047 |
|  |  | *DIABLO* | ENSBTAG00000004199 | 17 | 53155700-53173948 | 73677 |
|  |  | *B3GNT4* | ENSBTAG00000004197 | 17 | 53174272-53175345 | 72280 |
|  |  | *LRRC43* | ENSBTAG00000004192 | 17 | 53184647-53194577 | 53048 |
|  |  | *MLXIP* | ENSBTAG00000004189 | 17 | 53223788-53284407 | within |
|  |  | *BCL7A* | ENSBTAG00000032517 | 17 | 53304776-53332032 | -57151 |
|  |  | *U2* | ENSBTAG00000043751 | 17 | 53316080-53316260 | -68455 |
|  |  | *CFAP251* | ENSBTAG00000004187 | 17 | 53347984-53421121 | -100359 |
|  |  | *PSMD9* | ENSBTAG00000004179 | 17 | 53423935-53439245 | -176310 |
|  | rs132711282 | *RNF19A* | ENSBTAG00000017833 | 14 | 64047857-64103385 | 132147 |
|  |  | *SPAG1* | ENSBTAG00000032544 | 14 | 64174147-64208187 | 27345 |
|  |  | *POLR2K* | ENSBTAG00000022539 | 14 | 64216926-64220642 | 14890 |
|  |  | *FBXO43* | ENSBTAG00000019795 | 14 | 64227507-64239663 | within |
|  |  | *RGS22* | ENSBTAG00000019793 | 14 | 64289853-64398324 | -54321 |
|  | rs43496186 | *ARF1* | ENSBTAG00000007725 | 7 | 3001505-3019569 | 187604 |
|  |  | *WNT3A* | ENSBTAG00000039397 | 7 | 3035810-3087361 | 119812 |
|  |  | *WNT9A* | ENSBTAG00000020267 | 7 | 3155441-3163239 | 43934 |
|  |  | *PRSS38* | ENSBTAG00000013977 | 7 | 3253358-3285153 | -46185 |
|  |  | *SNAP47* | ENSBTAG00000004188 | 7 | 3403573-3469381 | -196400 |
|  | rs109425744 | *ITGA11* | ENSBTAG00000008380 | 10 | 15129322-15262276 | 119940 |
|  |  | *CORO2B* | ENSBTAG00000012681 | 10 | 15419306-15567397 | -37090 |
| MPY | rs109957491 | *MFSD1* | ENSBTAG00000009263 | 1 | 108706429-108732991 | -39241 |
|  |  | *RARRES1* | ENSBTAG00000014713 | 1 | 108807496-108849597 | -140308 |
|  |  | *GFM1* | ENSBTAG00000027795 | 1 | 108852511-108903175 | -185323 |
|  |  | *LXN* | ENSBTAG00000009336 | 1 | 108867031-108874756 | -199843 |
|  | rs109097262 | *PLCB4* | ENSBTAG00000013116 | 13 | 2304288-2503503 | -287691 |
|  | rs41906111 | *SPACA3* | ENSBTAG00000000344 | 19 | 17238257-17247945 | 74577 |
|  |  | *TMEM98* | ENSBTAG00000008913 | 19 | 17287825-17300526 | 21996 |
|  |  | *MYO1D* | ENSBTAG00000015527 | 19 | 17330801-17691005 | -8279 |
|  |  | *U6* | ENSBTAG00000049850 | 19 | 17432789-17432895 | -110267 |

*CHR: chromosome; MY: milk yield, MFP: milk fat percentage, MFY: milk fat yield, MPP: milk protein percentage, MPY: milk protein yield; The negative sign indicates that the SNP is in the upstream of the gene, and the positive sign indicates that the SNP is in the downstream of the gene.*
